# Supplementary material for: Hybrid contour and geometric partitioning for accurate plantar foot region segmentation
Source: PeerJ. 2025 Nov 20;13:e20352. doi: 10.7717/peerj.20352 (PMC12640632; doi:10.7717/peerj.20352)

In [2]:

```
'''
paper 1.1 comparison of three image segmentation methods.
'''
import cv2
import numpy as np
import pandas as pd
import os
import matplotlib.pyplot as plt

def apply_threshold_segmentation(image):
    # global thresholding
    _,threshSimple = cv2.threshold(image,127,255,cv2.THRESH_BINARY)
    # Otsu's thresholding
    _,threshOtsu = cv2.threshold(image,0,255,cv2.THRESH_BINARY+cv2.THR
    # Adaptive Thresholding
    threshAdaptive = cv2.adaptiveThreshold(image,255,cv2.ADAPTIVE_THRE
    return threshSimple,threshOtsu,threshAdaptive

def apply_canny_segmentation(image):
    # Apply edge-based segmentation
    edges100 = cv2.Canny(image, 100, 200)
    edges200 = cv2.Canny(image, 200, 400)
    edges300 = cv2.Canny(image, 300, 600)

    return edges100,edges200, edges300

def region_split_merge_segmentation(image):

    def Division_Judge(img, h0, w0, h, w) :
        # Function to determine if a region should be split
        area = img[h0 : h0 + h, w0 : w0 + w]
        mean = np.mean(area)
        std = np.std(area, ddof = 1)

        total_points = 0
        operated_points = 0

        for row in range(area.shape[0]) :
            for col in range(area.shape[1]) :
                if (area[row][col] - mean) < 2 * std :
                    operated_points += 1
                total_points += 1

        if operated_points / total_points >= 0.95 :
            return True
        else :
            return False

    def Merge(img, h0, w0, h, w, thresholds):
```

```
        segmented_img = img.copy()
        minThresh, maxThresh = thresholds  # Unpack the thresholds tup
        for row in range(h0, h0 + h):
            for col in range(w0, w0 + w):
                if img[row, col] > minThresh and img[row, col] < maxTh
                    segmented_img[row, col] = 0
                else:
                    segmented_img[row, col] = 255
        return segmented_img

    def Recursion(img, h0, w0, h, w, thresholds) :
        # Recursive function to split or merge regions based on certai
        if not Division_Judge(img, h0, w0, h, w) and min(h, w) > 5 :
            # Split the image into four quadrants and check for furthe
            Division_Judge(img, h0, w0, int(h0 / 2), int(w0 / 2))
            Division_Judge(img, h0, w0 + int(w0 / 2), int(h0 / 2), int
            Division_Judge(img, h0 + int(h0 / 2), w0, int(h0 / 2), int
            Division_Judge(img, h0 + int(h0 / 2), w0 + int(w0 / 2), in
        else :
            # Merge the regions
            return Merge(img, h0, w0, h, w, thresholds)

    img_gray = cv2.cvtColor(image, cv2.COLOR_BGR2GRAY)
    thresholds_list = [(50, 100), (100, 200), (200, 300)]  # Example t
    segmented_images = []
    for thresholds in thresholds_list:
        segmented_img = img_gray.copy()
        segmented_img = Recursion(segmented_img, 0, 0, segmented_img.s
                                  segmented_img.shape[1], thresholds)
        segmented_images.append(segmented_img)

    return segmented_images

def show_images(imgray, segImg1, segImg2,segImg3,titles):
    images = [imgray, segImg1, segImg2,segImg3]
    plt.figure(figsize=(12, 6))
    for i in range(4):
        plt.subplot(2,3,i+1)
        plt.imshow(images[i],'gray')
        plt.title(titles[i])
    plt.tight_layout()
    plt.show()

def compare_segmentation_results(image_path):
    image = cv2.imread(image_path)
    imgray = cv2.cvtColor(image, cv2.COLOR_BGR2GRAY)

    # Apply threshold segmentation algorithm
    threshSimple,threshOtsu,threshAdaptive = apply_threshold_segmentat
    titles=['Gray Image','threshSimple','threshOtsu','threshAdaptive']
    show_images(imgray, threshSimple, threshOtsu, threshAdaptive,title
```

```python
    # Apply region splitting and merging segmentation algorithm
    segImg_spli = region_split_merge_segmentation(image)
    titles=['Gray Image','split & merge (50, 100)','split & merge (100
    show_images(imgray, segImg_spli[0], segImg_spli[1], segImg_spli[2]

    # Apply canny segmentation algorithm
    canny100, canny200, canny300 = apply_canny_segmentation(imgray)
    titles=['Gray Image','canny (100,200)','canny (200, 400)','canny (
    show_images(imgray, canny100, canny200, canny300,titles)

if __name__ == '__main__':
    filename = './files/compareReg.csv'
    image_path = './images/normal/normal3.png'
    name = 'normal4.png'
    compare_segmentation_results(image_path)
```

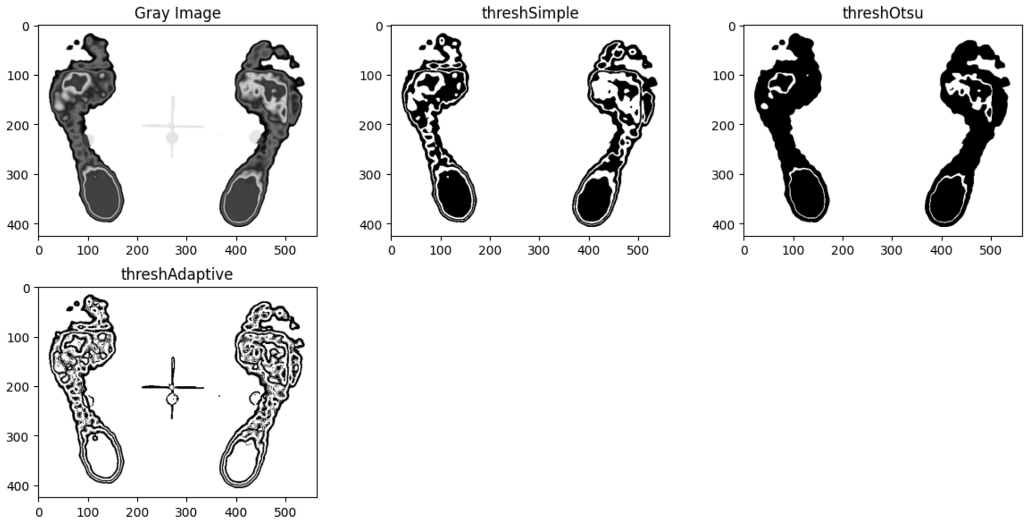

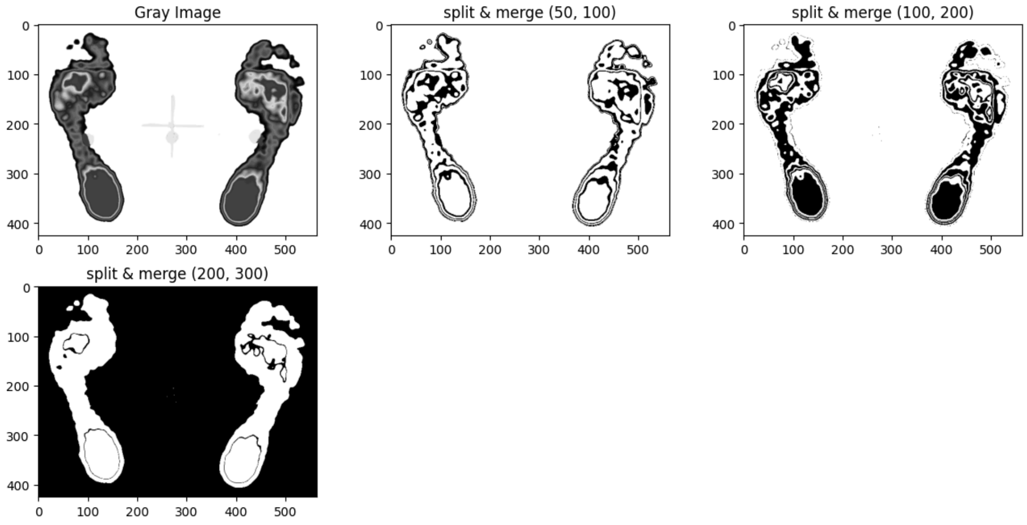

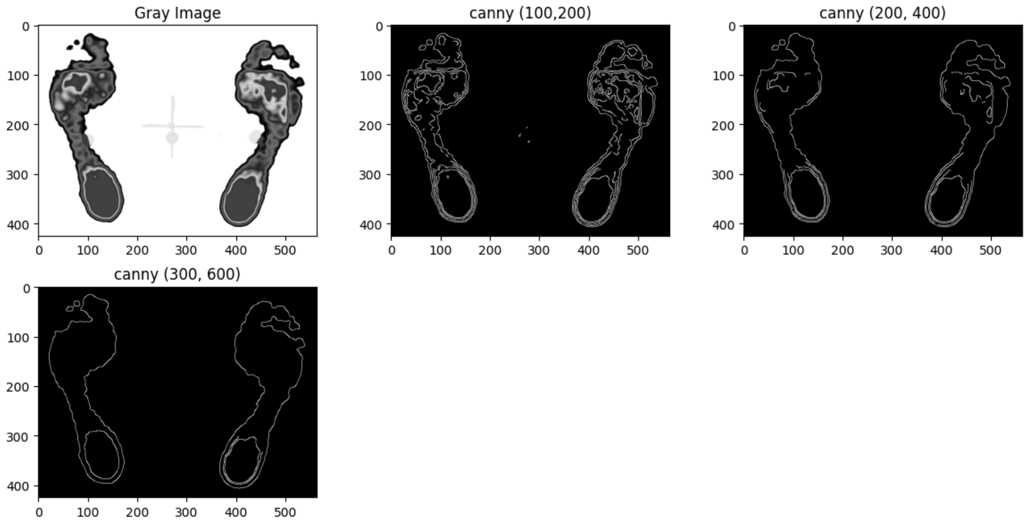

In [7]:

```
'''
paper 1.2 allContours VS. filteredContours.
'''
import cv2
import numpy as np
import pandas as pd
import os
import matplotlib.pyplot as plt

def apply_threshold_segmentation(image):
    _, threshold_image = cv2.threshold(cv2.cvtColor(image, cv2.COLOR_B
    # threshold_image = cv2.adaptiveThreshold(cv2.cvtColor(image, cv2.
    return threshold_image

def apply_canny_segmentation(image):
    # Apply Gaussian blur for denoising
    imageGaussian = cv2.GaussianBlur(image, (5, 5), 0)

    # Convert the image to grayscale
    imgray = cv2.cvtColor(imageGaussian, cv2.COLOR_BGR2GRAY)

    # Apply edge-based segmentation
    edges300 = cv2.Canny(cv2.cvtColor(image, cv2.COLOR_BGR2GRAY), 300,
    return edges300

def region_split_merge_segmentation(image):
    def Division_Judge(img, h0, w0, h, w) :
        area = img[h0 : h0 + h, w0 : w0 + w]
        mean = np.mean(area)
        std = np.std(area, ddof = 1)
        total_points = 0
        operated_points = 0

        for row in range(area.shape[0]) :
```

```
            for col in range(area.shape[1]) :
                if (area[row][col] - mean) < 2 * std :
                    operated_points += 1
                total_points += 1

        if operated_points / total_points >= 0.95 :
            return True
        else :
            return False

    def Merge(img, h0, w0, h, w) :
        for row in range(h0, h0 + h) :
            for col in range(w0, w0 + w) :
                if img[row, col] > 50 and img[row, col] < 100:
                    img[row, col] = 0
                else :
                    img[row, col] = 255

    def Recursion(img, h0, w0, h, w) :
        if not Division_Judge(img, h0, w0, h, w) and min(h, w) > 5 :
            Division_Judge(img, h0, w0, int(h0 / 2), int(w0 / 2))
            Division_Judge(img, h0, w0 + int(w0 / 2), int(h0 / 2), int
            Division_Judge(img, h0 + int(h0 / 2), w0, int(h0 / 2), int
            Division_Judge(img, h0 + int(h0 / 2), w0 + int(w0 / 2), in
        else :
            Merge(img, h0, w0, h, w)

    img_gray = cv2.cvtColor(image, cv2.COLOR_BGR2GRAY)
    segmented_img = img_gray.copy()
    Recursion(segmented_img, 0, 0, segmented_img.shape[0], segmented_i
    return segmented_img

def save_contours_to_csv(contours, method, filename,image, segImage):
    data = []
    interested_contours = []
    for i, contour in enumerate(contours):
        area = cv2.contourArea(contour)
        if area > 7000:
            interested_contours.append(contour)
            x, y, w, h = cv2.boundingRect(contour)
            data.append({'method': method, 'contour': i,'x': x,'y': y,
            print("Coordinates (x, y, w, h):", x, y, w, h)
            print(f"Contour {i + 1}: Area = {area}")
    df = pd.DataFrame(data)

    if not os.path.isfile(filename):
        df.to_csv(filename, index=False)
    else:  # Append to existing file
        df.to_csv(filename, mode='a', header=False, index=False)

    # Draw interested contours
```

```python
    contour_img = cv2.drawContours(image.copy(), interested_contours,
    allContour_img= cv2.drawContours(image.copy(),contours, -1, (51,25

    # Plot original and thresholded images
    plt.figure(figsize=(12, 6))
    plt.subplot(1, 3, 1)
    plt.imshow(cv2.cvtColor(image,cv2.COLOR_BGR2RGB))
    plt.title('Original image')

    plt.subplot(1,3,2)
    plt.imshow(cv2.cvtColor(allContour_img, cv2.COLOR_BGR2RGB))
    plt.title('allContours based on '+ method)

    plt.subplot(1, 3, 3)
    plt.imshow(cv2.cvtColor(contour_img, cv2.COLOR_BGR2RGB))
    plt.title('filteredContours based on '+ method)

    plt.tight_layout()
    plt.show()

def compare_segmentation_results(image_path):
    image = cv2.imread(image_path)

    # Apply threshold segmentation algorithm
    segImg_thre = apply_threshold_segmentation(image)
    threshold_contours, hierarchy = cv2.findContours(segImg_thre, cv2.
    save_contours_to_csv(threshold_contours, 'threshSimple', filename,
    # print(f'{threshold_contours=}')

    # Apply region splitting and merging segmentation algorithm
    segImg_spli = region_split_merge_segmentation(image)
    region_contours, hierarchy = cv2.findContours(segImg_spli, cv2.RET
    save_contours_to_csv(region_contours, 'split&merge (50, 100)', fil

    # Apply canny segmentation algorithm
    segImg_canny300 = apply_canny_segmentation(image)

    canny_contours300, hierarchy = cv2.findContours(segImg_canny300, c
    save_contours_to_csv(canny_contours300, 'canny (300, 600)', filena

if __name__ == '__main__':
    filename = './files/compareReg.csv'
    image_path = './images/normal/normal3.png'
    name = 'normal3.png'
    compare_segmentation_results(image_path)
```

```
Coordinates (x, y, w, h): 0 0 564 425
Contour 1: Area = 238712.0
Coordinates (x, y, w, h): 365 30 174 378
Contour 3: Area = 30980.0
Coordinates (x, y, w, h): 369 35 160 369
Contour 7: Area = 22115.0
Coordinates (x, y, w, h): 370 248 102 156
Contour 8: Area = 8556.0
Coordinates (x, y, w, h): 401 91 128 135
Contour 14: Area = 9708.0
Coordinates (x, y, w, h): 21 16 154 383
Contour 26: Area = 28525.0
Coordinates (x, y, w, h): 26 42 144 353
Contour 30: Area = 12224.5
Coordinates (x, y, w, h): 27 91 126 120
Contour 33: Area = 8636.5
```

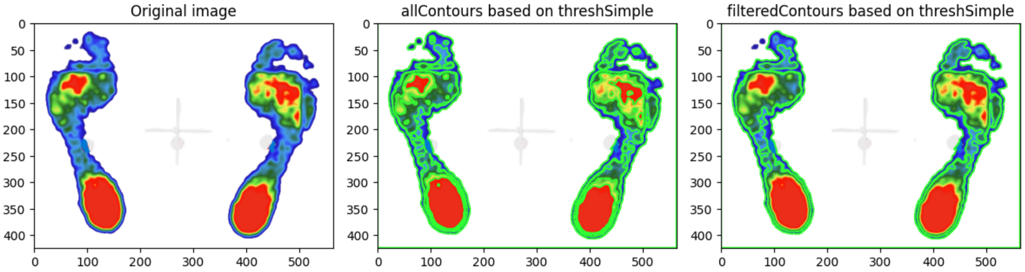


```
Coordinates (x, y, w, h): 84 269 85 125
Contour 81: Area = 7256.5
Coordinates (x, y, w, h): 402 92 105 112
Contour 224: Area = 8066.5
Coordinates (x, y, w, h): 369 34 161 371
Contour 304: Area = 23243.0
Coordinates (x, y, w, h): 365 30 172 378
Contour 312: Area = 29396.5
Coordinates (x, y, w, h): 26 23 145 372
Contour 318: Area = 22156.5
Coordinates (x, y, w, h): 21 16 154 383
Contour 321: Area = 27621.5
Coordinates (x, y, w, h): 0 0 564 425
Contour 322: Area = 238712.0
```

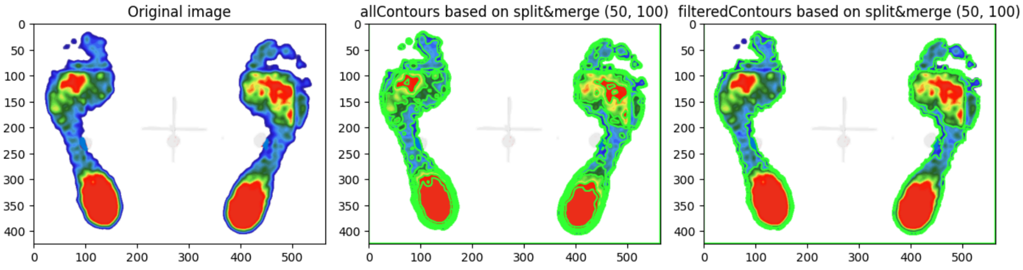

```
Coordinates (x, y, w, h): 366 31 172 377
Contour 3: Area = 30387.0
Coordinates (x, y, w, h): 366 31 172 377
Contour 4: Area = 30137.5
Coordinates (x, y, w, h): 22 17 152 381
Contour 9: Area = 27984.0
Coordinates (x, y, w, h): 22 17 152 381
Contour 10: Area = 27809.5
```

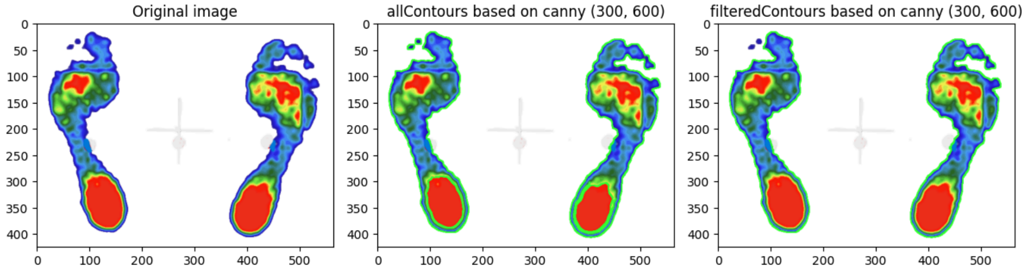

In [15]:

```
'''
paper 2.1 comparison of plantar image segmentation before and after fi
'''
import cv2
import numpy as np
import pandas as pd
import os
import matplotlib.pyplot as plt

def apply_threshold_segmentation(image):
    _, threshold_image = cv2.threshold(cv2.cvtColor(image, cv2.COLOR_B
    return threshold_image

def apply_canny_segmentation(image):
    # Apply Gaussian blur for denoising
    imageGaussian = cv2.GaussianBlur(image, (5, 5), 0)
    # Convert the image to grayscale
    imgray = cv2.cvtColor(imageGaussian, cv2.COLOR_BGR2GRAY)
    # Apply edge-based segmentation
    edges300 = cv2.Canny(cv2.cvtColor(image, cv2.COLOR_BGR2GRAY), 300,
    return edges300

def region_split_merge_segmentation(image):
    def Division_Judge(img, h0, w0, h, w) :
        area = img[h0 : h0 + h, w0 : w0 + w]
        mean = np.mean(area)
        std = np.std(area, ddof = 1)
        total_points = 0
        operated_points = 0
        for row in range(area.shape[0]) :
            for col in range(area.shape[1]) :
                if (area[row][col] - mean) < 2 * std :
                    operated_points += 1
```

```
                total_points += 1

        if operated_points / total_points >= 0.95 :
            return True
        else :
            return False

    def Merge(img, h0, w0, h, w) :
        for row in range(h0, h0 + h) :
            for col in range(w0, w0 + w) :
                if img[row, col] > 50 and img[row, col] < 100:
                    img[row, col] = 0
                else :
                    img[row, col] = 255

    def Recursion(img, h0, w0, h, w) :
        if not Division_Judge(img, h0, w0, h, w) and min(h, w) > 5 :
            Division_Judge(img, h0, w0, int(h0 / 2), int(w0 / 2))
            Division_Judge(img, h0, w0 + int(w0 / 2), int(h0 / 2), int
            Division_Judge(img, h0 + int(h0 / 2), w0, int(h0 / 2), int
            Division_Judge(img, h0 + int(h0 / 2), w0 + int(w0 / 2), in
        else :
            Merge(img, h0, w0, h, w)

    img_gray = cv2.cvtColor(image, cv2.COLOR_BGR2GRAY)
    segmented_img = img_gray.copy()
    Recursion(segmented_img, 0, 0, segmented_img.shape[0], segmented_i
    return segmented_img

def save_contours_to_csv(contours, method, filename, image, segImage):
    data = []
    interested_contours = []

    for i, contour in enumerate(contours):
        area = cv2.contourArea(contour)
        if area > 3000:
            interested_contours.append(contour)
            x, y, w, h = cv2.boundingRect(contour)
            data.append({'method': method, 'contour': i, 'x': x, 'y':
            print("Coordinates (x, y, w, h):", x, y, w, h)
            print(f"Contour {i + 1}: Area = {area}")

    df = pd.DataFrame(data)
    if not os.path.isfile(filename):
        df.to_csv(filename, index=False)
    else:
        df.to_csv(filename, mode='a', header=False, index=False)

    # Plot original and thresholded images
    plt.figure(figsize=(15, 5))
    plt.subplot(1, 3, 1)
```

```
    plt.imshow(cv2.cvtColor(image,cv2.COLOR_BGR2RGB))
    plt.title('Original image')

    plt.subplot(1, 3, 2)
    allContour_rectangles = draw_rectangles(contours, image.copy())
    plt.imshow(cv2.cvtColor(allContour_rectangles, cv2.COLOR_BGR2RGB))
    plt.title('Regions Rect. based on ' + method + ' allContours')

    plt.subplot(1, 3, 3)
    image_with_rectangles = draw_rectangles(interested_contours, image
    plt.imshow(cv2.cvtColor(image_with_rectangles, cv2.COLOR_BGR2RGB))
    plt.title('Regions Rect. based on '+ method + ' filteredContours')

    plt.tight_layout()
    plt.show()

def draw_rectangles(contours, image):
    for contour in contours:
        x, y, w, h = cv2.boundingRect(contour)
        cv2.rectangle(image, (x, y), (x + w, y + h), (0, 255, 0), 2)
    return image

def compare_segmentation_results(image_path):
    image = cv2.imread(image_path)

    # Apply threshold segmentation algorithm
    segImg_thre = apply_threshold_segmentation(image)
    threshold_contours, hierarchy = cv2.findContours(segImg_thre, cv2.
    save_contours_to_csv(threshold_contours, 'threshSimple', filename,
    # print(f'{threshold_contours=}')

    # Apply region splitting and merging segmentation algorithm
    segImg_spli = region_split_merge_segmentation(image)
    region_contours, hierarchy = cv2.findContours(segImg_spli, cv2.RET
    save_contours_to_csv(region_contours, 'Split & Merge', filename,im

    # Apply canny segmentation algorithm
    segImg_canny300 = apply_canny_segmentation(image)

    canny_contours300, hierarchy = cv2.findContours(segImg_canny300, c
    save_contours_to_csv(canny_contours300, 'Canny (300,600)', filenam

if __name__ == '__main__':
    filename = './files/compareReg.csv'
    image_path = './images/lowarch/lowarch12.png'
    name = 'lowarch12.png'
    compare_segmentation_results(image_path)
```

```
Coordinates (x, y, w, h): 0 0 518 391
Contour 1: Area = 201630.0
Coordinates (x, y, w, h): 9 63 140 318
Contour 2: Area = 28300.5
Coordinates (x, y, w, h): 31 193 110 180
Contour 21: Area = 9088.0
Coordinates (x, y, w, h): 54 268 85 103
Contour 22: Area = 5637.5
Coordinates (x, y, w, h): 359 49 152 321
Contour 32: Area = 28741.0
Coordinates (x, y, w, h): 367 71 133 294
Contour 48: Area = 18403.5
Coordinates (x, y, w, h): 369 196 114 167
Contour 50: Area = 7432.0
```

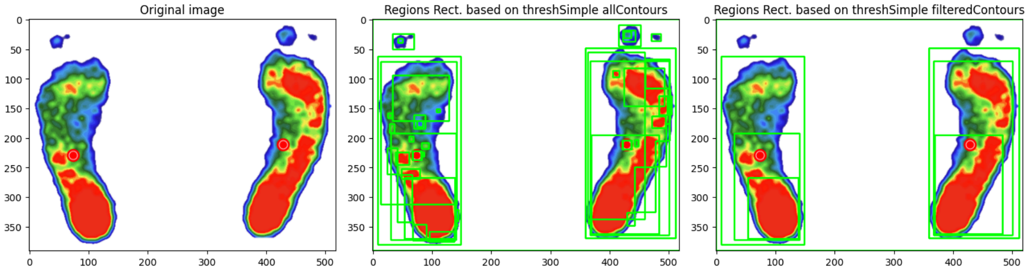


```
Coordinates (x, y, w, h): 73 287 62 79
Contour 40: Area = 3537.0
Coordinates (x, y, w, h): 374 279 66 79
Contour 41: Area = 3323.0
Coordinates (x, y, w, h): 55 269 84 101
Contour 48: Area = 5401.0
Coordinates (x, y, w, h): 370 197 112 165
Contour 97: Area = 7112.5
Coordinates (x, y, w, h): 32 93 97 86
Contour 165: Area = 3342.5
Coordinates (x, y, w, h): 16 86 116 290
Contour 169: Area = 8308.0
Coordinates (x, y, w, h): 10 65 129 292
Contour 200: Area = 3006.0
Coordinates (x, y, w, h): 364 63 138 297
Contour 202: Area = 3083.0
Coordinates (x, y, w, h): 0 0 518 391
Contour 251: Area = 201630.0
```

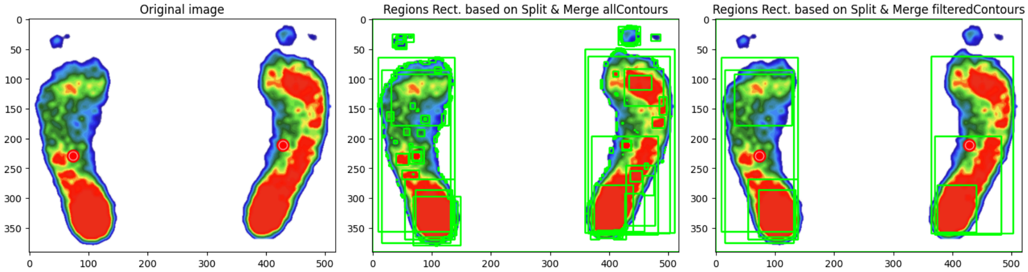

```
Coordinates (x, y, w, h): 9 64 140 316
Contour 1: Area = 28217.0
Coordinates (x, y, w, h): 9 64 140 316
Contour 2: Area = 28011.0
Coordinates (x, y, w, h): 359 50 151 320
Contour 14: Area = 28748.5
Coordinates (x, y, w, h): 359 50 151 320
Contour 15: Area = 28522.5
```

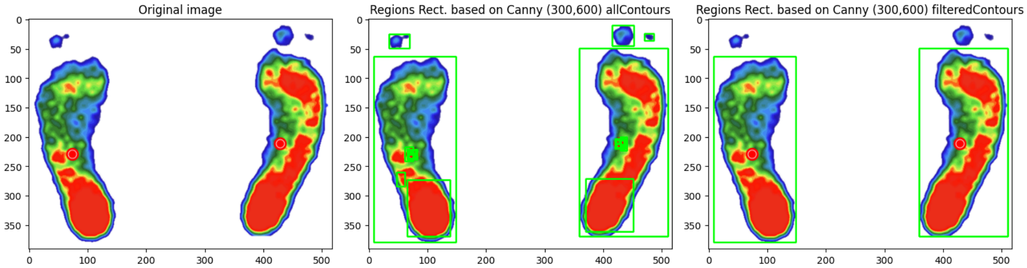

Supplement: Supplemental Information 1 [file peerj-13-20352-s001.pdf]
