# Supplementary material for: Hybrid contour and geometric partitioning for accurate plantar foot region segmentation
Source: PeerJ. 2025 Nov 20;13:e20352. doi: 10.7717/peerj.20352 (PMC12640632; doi:10.7717/peerj.20352)
Supplement: Supplemental Information 2 [file peerj-13-20352-s002.pdf]

In [2]:

```python
# experiments: combined segmentation & pressure distribution
import cv2
import matplotlib.pyplot as plt
import csv
import numpy as np

def get_color_name_hsv(hsv_color):
    """Returns the name of the color based on its HSV values."""
    hue, saturation, value = hsv_color
    if 25 <= saturation <= 255 and 50 <= value <= 255:
        if 0 <= hue <= 10 or 156 <= hue <= 180:
            return "Red" if 46 <= value <= 220 else "Light Red"
        elif 11 <= hue <= 25:
            return "Orange" if 43 <= saturation <= 255 else "Light Ora
        elif 26 <= hue <= 34:
            return "Yellow" if 43 <= saturation <= 255 else "Light Yel
        elif 35 <= hue <= 77:
            return "Green" if 43 <= saturation <= 255 else "Light Gree
        elif 78 <= hue <= 99:
            return "Cyan" if 43 <= saturation <= 255 else "Light Cyan"
        elif 100 <= hue <= 124:
            return "Blue" if 46 <= value <= 255 else "Light Blue"
        elif 125 <= hue <= 155:
            return "Purple" if 46 <= value <= 255 else "Light Purple"
    elif 0 <= saturation <= 50 and 0 <= value <= 200:
        return "Gray" if value < 201 else "Dark Gray"
    elif 0 <= saturation <= 50 and 0 <= value <= 50:
        return "Black" if hue <= 180 else "Light Black"
    elif 0 <= saturation <= 50 and 200 <= value <= 255:
        return "White" if hue <= 180 else "Light White"
    return "Unknown"

def save_pixelColor(image):
    hsv_image = cv2.cvtColor(image, cv2.COLOR_BGR2HSV)
    height, width, _ = image.shape

    pixelCoord_color = {}
    for y in range(height):
        for x in range(width):
            color = hsv_image[y, x]
            color_name = get_color_name_hsv(color)
            if color_name != 'White':
                pixelCoord_color[(x, y)] = color_name

    return pixelCoord_color

def contours_imgSegm(image, width):
    # Convert the image to grayscale
    imgray = cv2.cvtColor(image, cv2.COLOR_BGR2GRAY)
```

```
# Apply edge-based segmentation
edges300 = cv2.Canny(imgray, 300, 600)

# Find contours from the edge image
contours, _ = cv2.findContours(edges300, cv2.RETR_EXTERNAL, cv2.CH

# Get the dimensions of the image
height, width = edges300.shape[:2]
print(f'{height=},{width=}')
# Calculate the center point of the image
center_x = width // 2

# Split the image into leftFoot and rightFoot
leftFoot = edges300[:, :center_x-40]
rightFoot = edges300[:, center_x:]

# Calculate minLeft_y, maxLeft_y for leftFoot
left_foot_edges = leftFoot

# Find the indices of non-zero elements (edge pixels) in the left
left_nonzero_indices_x = np.nonzero(left_foot_edges)[1]

# Calculate min and max x values for left foot
minLeft_x = np.min(left_nonzero_indices_x)
maxLeft_x = np.max(left_nonzero_indices_x)

# Find the indices of non-zero elements (edge pixels) in the left
left_nonzero_indices = np.nonzero(left_foot_edges)

# Calculate min and max y values for left foot
minLeft_y = np.min(left_nonzero_indices[0])
maxLeft_y = np.max(left_nonzero_indices[0])

print(f'{minLeft_x=},{maxLeft_x=},{minLeft_y=},{maxLeft_y=}')

if toe == 0:
    minLy= minLeft_y+20
else:
    minLy=minLeft_y+int(maxLeft_y*0.16)

leftY = maxLeft_y - minLy + 1
leftY_fron,leftY_arch,leftY_heel = int(leftY*0.30),int(leftY*0.30)

# Find the indices of x values within each region
front_indices = np.where((left_nonzero_indices[0] >= minLy) & \
                     (left_nonzero_indices[0] <= (minLy + leftY_fr
arch_indices = np.where((left_nonzero_indices[0] > (minLy + leftY_
              (left_nonzero_indices[0] <= (minLy + leftY_fron + left
heel_indices = np.where((left_nonzero_indices[0] > (maxLeft_y-left
                    (left_nonzero_indices[0] <= maxLeft_y))[0]
```

```python
# Calculate min and max x values for each region in leftFoot
minF_x = np.min(left_nonzero_indices_x[front_indices])
maxF_x = np.max(left_nonzero_indices_x[front_indices])

minA_x = np.min(left_nonzero_indices_x[arch_indices])
maxA_x = np.max(left_nonzero_indices_x[arch_indices])

minH_x = np.min(left_nonzero_indices_x[heel_indices])
maxH_x = np.max(left_nonzero_indices_x[heel_indices])

fourRegions=[]
fourRegions.append(('left_outerForefoot',(minF_x, minLy),\
                (minF_x+(maxF_x-minF_x)//2,minLy+leftY_fron)))
fourRegions.append(('left_innerForefoot',(minF_x+(maxF_x-minF_x)//
                (maxF_x, minLy+leftY_fron)))
fourRegions.append(('left_arch',(minA_x,minLy+leftY_fron),\
                (maxA_x, minLy+leftY_fron+leftY_arch)))
fourRegions.append(('left_heel',(minH_x,minLy+leftY_fron+leftY_arc
                (maxH_x,maxLeft_y)))

# Calculate minRight_y, maxRight_y for rightFoot
right_foot_edges = rightFoot

# Find the indices of non-zero elements (edge pixels) in the right
right_nonzero_indices_x = np.nonzero(right_foot_edges)[1]

# Calculate min and max x values for left foot
minRight_x = np.min(right_nonzero_indices_x)+center_x
maxRight_x = np.max(right_nonzero_indices_x)+center_x

# Find the indices of non-zero elements (edge pixels) in the right
right_nonzero_indices = np.nonzero(right_foot_edges)

# Calculate min and max y values for right foot
minRight_y = np.min(right_nonzero_indices[0])
maxRight_y = np.max(right_nonzero_indices[0])

print(f'{minRight_x=},{maxRight_x=},{minRight_y=},{maxRight_y=}')

if toe == 0:
    minRy= minRight_y +60
else:
    minRy=minRight_y+int(maxRight_y*0.16)

rightY = maxRight_y - minRy + 1
# rightY_fron,rightY_arch,rightY_heel = int(rightY*0.36),int(right
rightY_fron,rightY_arch,rightY_heel = int(rightY*0.30),int(rightY*

# Find the indices of x values within each region
front_indicesR = np.where((right_nonzero_indices[0] >= minRy) & \
                     (right_nonzero_indices[0] <= (minRy + rightY_
```

```
    arch_indicesR = np.where((right_nonzero_indices[0] > (minRy + righ
                (right_nonzero_indices[0] <= (minRy + rightY_fron + ri
    heel_indicesR = np.where((right_nonzero_indices[0] > (maxRight_y-r
                        (right_nonzero_indices[0] <= maxRight_y))[0]

    # Calculate min and max x values for each region in rightFoot
    minF_xR = np.min(right_nonzero_indices_x[front_indicesR])+center_x
    maxF_xR = np.max(right_nonzero_indices_x[front_indicesR])+center_x

    if right_nonzero_indices_x[arch_indicesR].size > 0:
        minA_xR = np.min(right_nonzero_indices_x[arch_indicesR])+cente
        maxA_xR = np.max(right_nonzero_indices_x[arch_indicesR])+cente
    else:
        minA_xR = minF_xR+20
        maxA_xR = maxF_xR-20

    minH_xR = np.min(right_nonzero_indices_x[heel_indicesR])+center_x
    maxH_xR = np.max(right_nonzero_indices_x[heel_indicesR])+center_x

    # Append the four regions to the fourRegions list
    fourRegions.append(('right_innerForefoot',(minF_xR,minRy), \
                (minF_xR +(maxF_xR-minF_xR)//2,minRy+rightY_fron)))
    fourRegions.append(('right_outerForefoot',(minF_xR +(maxF_xR-minF_
                                           (maxF_xR, minRy + rightY_fr
    fourRegions.append(('right_arch',(minA_xR,minRy+rightY_fron),\
                    (maxA_xR, minRy+rightY_fron+rightY_arch)))
    fourRegions.append(('right_heel',(minH_xR,minRy+rightY_fron+rightY
                        (maxH_xR,maxRight_y)))

    print(f'{fourRegions=}')

    return fourRegions,contours

def presCalc(regions, binImg, pixeColors, presColors):
    regiInfo = []
    leftPressure = 0
    rightPressure = 0
    # pixel pressure calculation
    for side, topLeft, botRight in regions:
        regiPixels = binImg[topLeft[1]:botRight[1] + 1, topLeft[0]:bot
        pressureCount = 0  # Initialize pressure_count dictionary

        # Iterate over each pixel in the region
        for y in range(regiPixels.shape[0]):
            for x in range(regiPixels.shape[1]):
                color_name = pixeColors.get((x + topLeft[0], y + topLe
                if color_name is not None:
                    pressure = presColors.get(color_name, 0)
                    pressureCount = pressureCount + pressure

        if side.split('_')[0] == 'left':    #side == 'left':
```

```
            leftPressure += pressureCount
        else:
            rightPressure += pressureCount

        regiInfo.append({
            'side': side,
            'topLeft': topLeft,
            'botRight': botRight,
            'pressureCount': pressureCount
        })
    return regiInfo, leftPressure, rightPressure

if __name__ == "__main__":
    img_name = "./images/out/inner14.png"  # image-cropped.png
    name="inner14.png"; toe=1
    image = cv2.imread(img_name)
    if image is None:
       print("Error: Image is empty")
    else:
       imgray = cv2.cvtColor(image, cv2.COLOR_BGR2GRAY)
    _, bin_img = cv2.threshold(imgray, 127, 255, cv2.THRESH_BINARY)

    # Convert the image to RGB format
    img_rgb = cv2.cvtColor(image, cv2.COLOR_BGR2RGB)

    h, w = bin_img.shape
    img=img_rgb

    pixeColors = save_pixelColor(image)
    presColors = {
        'Light Red': 240, 'Red': 220,
        'Light Orange': 195, 'Orange': 180,
        'Light Yellow': 170, 'Yellow': 130,
        'Light Green': 120, 'Green': 80,
        'light Cyan' : 50, 'Cyan': 40,
        'Light Blue': 30, 'Blue': 10,
        'Light Purple': 5, 'Purple': 3,
        'Gray': 0, 'White': 0, 'Black': 0,
        'Unknown': 1
    }

    regions, contours = contours_imgSegm(img, w)
    regiInfo, leftPressure, rightPressure = presCalc(regions, bin_img,

    for i, region in enumerate(regiInfo):
        side = region['side']
        topLeft = region['topLeft']
        botRight = region['botRight']
        pressureCount = region['pressureCount']
        if leftPressure + rightPressure != 0:
            weightPercentage = pressureCount / (leftPressure + rightPr
```

```
        else:
            weightPercentage = 0  # Assign a default value or handle i

        regiInfo[i]['weightPercentage'] = weightPercentage

        center_x = (topLeft[0] + botRight[0]) // 2
        center_y = (topLeft[1] + botRight[1] + 25) // 2
        # cv2.arrowedLine(img_rgb, (center_x, center_y), (center_x, ce
        cv2.putText(img, f"{weightPercentage:.0f}%", (center_x-10, cen
                    cv2.FONT_HERSHEY_SIMPLEX, 0.6, (0, 0, 0), 1)
        cv2.rectangle(img,(topLeft[0],topLeft[1]),(botRight[0], botRig

    # To calculate the bearing ratio for the left foot and right foot
    leftBearing_ratio = (leftPressure / (leftPressure + rightPressure)
    rightBearing_ratio = (rightPressure / (leftPressure + rightPressur

    # put the results as text in the image
    cv2.putText(img, f'leftBearing={leftBearing_ratio:.0f}%',(int(w/2-
                cv2.FONT_HERSHEY_SIMPLEX,0.5,(0,0,0),1)
    cv2.putText(img,f'rightBearing={rightBearing_ratio:.0f}%',(int(w/2
                cv2.FONT_HERSHEY_SIMPLEX,0.5,(0,0,0),1)
    cv2.putText(img,f'image={name}',(int(w/2-70),60),\
                cv2.FONT_HERSHEY_SIMPLEX,0.5,(0,0,0),1)

    # Draw the interested contours,(0, 255, 0)
    contour_img = cv2.drawContours(img.copy(), contours, -1, (0, 255,
    plt.imsave("./figures/out/"+name, contour_img)

    # Display the image with drawn contours
    plt.imshow(contour_img, cmap='gray')   #, cmap='gray'

    # Show the plot
    plt.show()

    ls=[{'side':'wholeFoot','leftBearing':leftBearing_ratio,'rightBear
    regiInfo += ls

    # Get all unique field names from the dictionaries
    fieldnames = set().union(*(d.keys() for d in regiInfo))

    # Specify the file name for the CSV
    filename = './files/regiInfoWhole.csv'

    with open(filename, mode='a', newline='') as file:
       writer = csv.writer(file)
       writer.writerow([img_name])

    # Open the file in write mode and create a CSV writer object
    with open(filename, mode='a', newline='') as file:
        writer = csv.DictWriter(file, fieldnames=fieldnames)
        # Write the header row
```

```
        writer.writeheader()

        # Write each dictionary as a row in the CSV file
        for row in regiInfo:
            writer.writerow(row)

    print("regiInfo saved successfully to", filename)
```

```
height=412,width=589
minLeft_x=9,maxLeft_x=190,minLeft_y=16,maxLeft_y=399
minRight_x=386,maxRight_x=577,minRight_y=9,maxRight_y=384
fourRegions=[('left_outerForefoot', (9, 79), (79, 175)), ('left_innerFo
refoot', (79, 79), (150, 175)), ('left_arch', (21, 175), (134, 271)),
('left_heel', (63, 271), (190, 399)), ('right_innerForefoot', (437, 7
0), (507, 164)), ('right_outerForefoot', (507, 70), (577, 164)), ('righ
t_arch', (441, 164), (573, 258)), ('right_heel', (386, 258), (522, 38
4))]
```

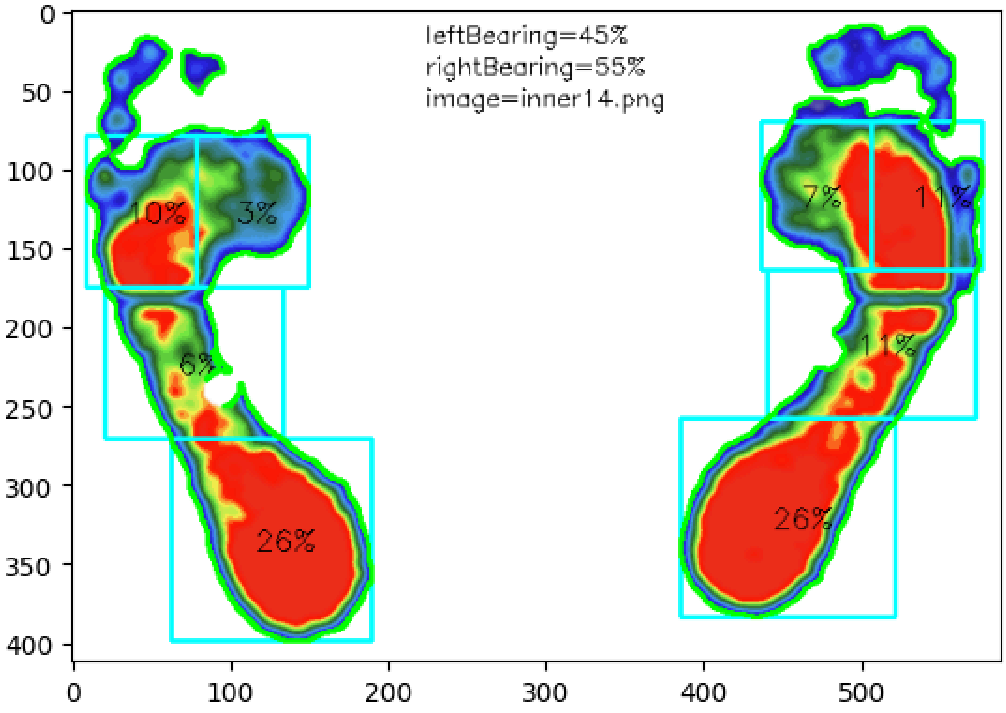


```
regiInfo saved successfully to ./files/regiInfoWhole.csv
```
